# AS-703026 Inhibits LPS-Induced TNFα Production through MEK/ERK Dependent and Independent Mechanisms

**DOI:** 10.1371/journal.pone.0137107

**Published:** 2015-09-18

**Authors:** Ping Li, Yonghong Wu, Manxiang Li, Xiaojuan Qiu, Xiaoyan Bai, Xiaojing Zhao

**Affiliations:** 1 Department of Emergency, the Second Affiliated Hospital of Xi'an Jiao Tong University, Xi'an, China; 2 Department of clinical immunology and pathogenic examination, Xi'an Medical University, Xi'an, China; 3 The First Affiliated Hospital of Xi'an Jiao Tong University, Xi'an, China; Suzhou University, CHINA

## Abstract

Chronic obstructive pulmonary disease (COPD) is characterized by intense lung infiltrations of immune cells (macrophages and monocytes). Lipopolysaccharide (LPS) activates macrophages/monocytes, leading to production of tumor necrosis factor α (TNFα) and other cytokines, which cause subsequent lung damages. In the current study, our results demonstrated that AS-703026, a novel MEK/ERK inhibitor, suppressed LPS-induced TNFα mRNA expression and protein secretion in RAW 264.7 murine macrophages, and in murine bone marrow-derived macrophages (BMDMs). Meanwhile, TNFα production in LPS-stimulated COPD patents’ peripheral blood mononuclear cells (PBMCs) was also repressed by AS-703026. At the molecular level, we showed that AS-703026 blocked LPS-induced MEK/ERK activation in above macrophages/monocytes. However, restoring ERK activation in AS-703026-treated RAW 264.7 cells by introducing a constitutive-actively (CA)-ERK1 only partially reinstated LPS-mediated TNFα production. Meanwhile, AS-703026 could still inhibit TNFα response in ERK1/2-depleted (by shRNA) RAW 264.7 cells. Significantly, we found that AS-703026 inhibited LPS-induced nuclear factor κB (NFκB) activation in above macrophages and COPD patients’ PBMCs. *In vivo*, oral administration of AS-703026 inhibited LPS-induced TNFα production and endotoxin shock in BALB/c mice. Together, we show that AS-703026 *in vitro* inhibits LPS-induced TNFα production in macrophages/monocytes, and *in vivo* protects mice from LPS-induced endotoxin shock. Thus, it could be further studied as a useful anti-inflammatory therapy for COPD patients.

## Introduction

Chronic obstructive pulmonary disease (COPD) is a major health problem in China and around the world. It is a progressive disorder characterized by massive airway inflammations [1,2,3]. Physiologically, COPD is accompanied with expiratory airflow obstruction, *i*.*e*. emphysema and chronic bronchitis [1,2,3]. Despite the fact that COPD’s incidence is increasing at an alarming rate, there has been no specific and/or effective therapies available to limit or prevent the progression or airway destruction of COPD [1,2,3]. Thus, there is an urgent need to explore novel agents targeting this disease.

Lung inflammation in COPD is often associated with an increased level of circulating pathogen-associated molecular patterns (PAMPs), leading to local infiltrations of macrophages and monocytes, which produce several pro-inflammatory cytokines, including tumor necrosis factor-α (TNFα), interleukin 1β (IL-1β) and many others [4,5]. One of the most prominent PAMPs is lipopolysaccharide (LPS) or endotoxin, which is a gram-negative bacteria wall product [6,7]. In COPD, LPS is known as an important inducer and contributor of lung inflammations and injuries [8,9]. Several anti-LPS strategies have displayed benefits against COPD inflammations [8,9].

Circulating LPS will be sensed by CD14 and LPS-binding protein (LBP), and binds to Toll-like receptor 4 (TLR-4) on the plasma surface of macrophages/monocytes [6,10]. After LPS-TLR-4 binding, a number of adaptor proteins [including myeloid differentiation factor 88 (MyD88), IL-1R-associated kinase (IRAK), and tumor necrosis factor receptor-associated factor 6 (TRAF6)] will be recruited to the receptor complex, leading to activation of nuclear factor κB (NFκB) and mitogen-activated protein kinases (MAPKs) signaling cascades [6,10]. Activation of these signalings mediates expression and production of various pro-inflammatory cytokines, including TNFα [6,10].

TNFα level is increased in bronchoalveolar lavage fluids, sputum, and plasma/lung tissues of COPD patients, serving as an important contributor of COPD lung injuries [9,11,12]. The extracellular signal-regulated kinase (ERK) kinase (MEK)/ERK kinase pathway is important for the activation of AP-1 and C/EBPβ transcription factors, required for the transcription of TNFα [13,14]. Pharmacological or genetic inhibition of MEK-ERK signaling was shown to inhibit TNFα production by LPS [13,14]. In this study, we investigated the potential effect of AS-703026, a novel MEK/ERK inhibitor [15,16,17], against LPS-induced TNFα production *in vitro* and endotoxin shock *in vivo*. The underlying signaling mechanisms were also investigated.

## Materials and Methods

### 2.1. Chemicals, reagents and antibodies

LPS, D-galactosamine, PD98059 and U0126 were obtained from Sigma (St. Louis, MO). Anti-MEK1/2, ERK1/2, β-tubulin and IKKα/β antibodies were purchased from Santa Cruz (Santa Cruz, CA). All other antibodies were obtained from Cell Signaling Technology (Danvers, MA). Cell culture reagents were purchased from Gibco (Shanghai, China).

### 2.2. RAW 264.7 mouse macrophage culture

RAW 264.7 cells were from Dr. Shen’s group [18], and were cultured in DMEM supplemented with 10% heat-inactivated FBS, 3 mM glutamine and necessary antibiotics [18].

### 2.3. Bone marrow—derived macrophages (BMDMs) culture

As reported [18], the bone marrow of C57/BL6 mice (two month old) was flushed. Cell pellets were resuspended in ACK hypotonic buffer, and were subsequently washed and cultured in RPMI supplemented with 10% FBS and 30% L-929 media. After 6–7 days, adherent macrophages were trypsinized, counted, re-plated for experimental use. All animal experiments were approved by the Second Affiliated Hospital of Xi'an Jiao Tong University Institutional Animal Care and Use Committee (IACUC, contact person: Dr. Ping Ling).

### 2.4. Ex-vivo culture of human peripheral blood mononuclear cells (PBMCs)

PBMCs of COPD patients (male, 52–65 years old) were collected by centrifugation over lymphocyte separation medium, and were washed in PBS. PBMCs were cultured in DMEM plus 15% FBS, and necessary supplements (see [19]). PBMCs were serum starved for experimental use. Experiments and the protocols requiring clinical samples were approved by the ethics committee of the Second Affiliated Hospital of Xi'an Jiao Tong University (contact person: Dr. Wei Liu). The written informed consent was obtained from each participant. A total of five COPD patients administrated in authors’ hospital were enrolled. All investigations were conducted according to the principles expressed in the Declaration of Helsinki.

### 2.5. MTT assay for cell viability

Cytotoxicity was analyzed by MTT (Sigma, Shanghai, China) assay. Briefly, macrophages/monocytes were plated at a density of 1×10^4^ cells/mL onto 96-well plates. After treatment, MTT (5 mg/mL, 20 μL/well) was added and further incubated for 4 h. Afterwards, cells were resolved with 150μL DMSO per well, followed by optical density (OD) measurement at 490 nm with a ELX800-UV absorbance microplate reader (BioTek Instruments Inc., Winooski, VT).

### 2.6. Cell death assay

After applied treatment, cell death was tested by trypan blue staining. Cells positive for trypan blue staining were considered dead [20].

### 2.7. TNFα enzyme-linked immunosorbent assay (ELISA)

After treatment, TNFα content in the conditional medium was measured with a TNFα ELISA kit (R&D Systems, Abingdon, UK) with the manufacturer’s instructions.

### 2.8. Total RNA isolation and real-time reverse transcriptase polymerase chain reaction (RT-PCR)

Total RNA was extracted by the RNA-TRIZOL reagents (Gibco, Shanghai, China). RT-PCR was performed by using TOYOBO ReverTra Ace RT-PCR kit with the manufacturer’s instructions. A typical reaction (50 μL) contained 1/50 of reverse transcription—generated cDNA and 200 nM of primer in 1× SYBR Green RealTime Master Mix (Toyobo, Shanghai, China) buffer. The PCR reactions were carried out on a Bio-Rad IQ5 multicolor detection system by using 2 μg of synthesized cDNA. Following primers were utilized: TNFα-forward: 5′-ATGAGCACTGAAAGCATGATC-3′; reverse: 5′-CAGATGACCTAGTAACGGACT-3′ [18,19]. Glyceraldehyde-3-phosphate dehydrogenase (GAPDH)-forward: 5'-CAATGACCCCTTCATTGACC-3'; reverse: 5'-GACAAGCTTCCCGTTCTCAG-3'. All real-time PCRs were performed at least in triplicate. The relative TNFα expression was calculated by the comparative Ct method (2^−ΔΔCt^) [21], using GAPDH as the reference gene. The TNFα mRNA expression level was expressed as the fold change vs. control group.

### 2.9. Western blots

Total cell lysates, denatured in sample buffer, were subjected to SDS—PAGE on 10% gel, and separated proteins were transferred onto PVDF membranes. Membranes were blocked with blocking buffer for 1 h at room temperature and, as desired, probed with primary antibodies against indicated kinases or the loading control overnight at 4°C followed by peroxidase-conjugated appropriate secondary antibodies and ECL detection. The bands were quantified by Bio-Rad Quantity One software. The intensities of the target bands were always normalized to corresponding equal loadings.

### 2.10. ERK1/2 shRNA and stable cell selection

The ERK1/2 short hairpin RNA (shRNA)-containing lentiviral particles (sc-29307-SH and sc-35335-SH) or the scramble non-sense control shRNA lentiviral particles (sc-108080) were obtained from Santa Cruz Biotech (Shanghai, China). The lentiviral particles (15 μL/mL) were added to macrophages for 48 h, and stable colonies expressing targeted-shRNA were selected by puromycin (0.25 μg/mL, Sigma) for 8 days. The expression of ERK1/2 was tested by Western blots in the resistant colonies.

### 2.11. Constitutively-active (CA) ERK1 plasmid and transfection

A constitutively-active (CA) ERK1 (T217D/Y221D)-puromycin-GFP plasmid was from Dr. Tong’s Lab [19]. Lipofectamine 2000 was applied to transfect CA-ERK1 plasmid or the empty vector [19] into RAW 264.7 cells. Stable cells were again selected by puromycin (0.25 μg/mL) for 8 days. After selection, more than 90% of RAW 264.7 cells were GFP positive, indicating a decent transfection efficiency.

### 2.12. Measuring NFκB (p65) DNA-binding activity

After treatment, nuclear proteins of macrophages/monocytes were extracted using commercially available reagents according to the manufacturer's instructions (Sigma, Shanghai, China). NFκB (p65) DNA-binding activity was examined using the TransAM™ ELISA kit (Active Motif, Carlsbad, CA) according to the manufacturer's protocol. In brief, 1.0 μg of nuclear extract was subjected to the binding of NFκB to an immobilized consensus sequence in a 96-well plate, and the primary and secondary antibodies were added. After the colorimetric reaction, OD value of samples was measured in an ELISA reader at 450 nm.

### 2.13. LPS-induced endotoxin shock

BALB/c mice were maintained *ad libitum* under the temperature of 24 ±1°C, humidity of 40–80%, and a 12-h light/12-h dark cycle. All the animal procedures were in accordance with the guide for the Care and Use of Laboratory Animals published by the US National Institutes of Health and authors’ institutions. Mice were injected intraperitoneally (*i*.*p*.) with LPS (30 mg/kg body weight) and D-galactosamine (300 mg/kg body weight) [19,22], the latter is a hepatotoxic transcriptional inhibitor which sensitizes the animals to the cytotoxic effects of TNF-α, or plus gastric lavage of AS-703026 (30 mg/kg mice body weight). Mice death was recorded 24 h and 48 h after LPS administration, tail vein serum samples were collected 4 h and 8 h after LPS stimulation, TNFα content was determined by ELISA using the same protocol described in [19]. The concentration of AS-703026 was chosen based on results of pre-experiments and previous publications [15,17]. For the survival studies, humane endpoints were applied to minimize suffering. Animals were observed and weighed after the original LPS/D-galactosamine administration every hour. Clinical signs of endotoxin shock included significant reduced locomotion, signs of severe diarrhea, piloerection and a body weight loss exceeding 20% of the initial body-weight. If animals reached these endpoints they were euthanized by exsanguination under anesthesia. All intraperitoneal injections were performed under anesthesia.

### 2.14. Data analysis

Data were collected using a minimum of three experiments and used to calculate the mean ± S.D. Statistical differences were analyzed by one-way *ANOVA* followed by multiple comparisons performed with post hoc Bonferroni test (SPSS version 18). Values of *p*<0.05 were considered statistically significant.

## Results

### 3.1. AS-703026 inhibits LPS-induced TNFα production in RAW 264.7 murine macrophages

**Fig 1:**
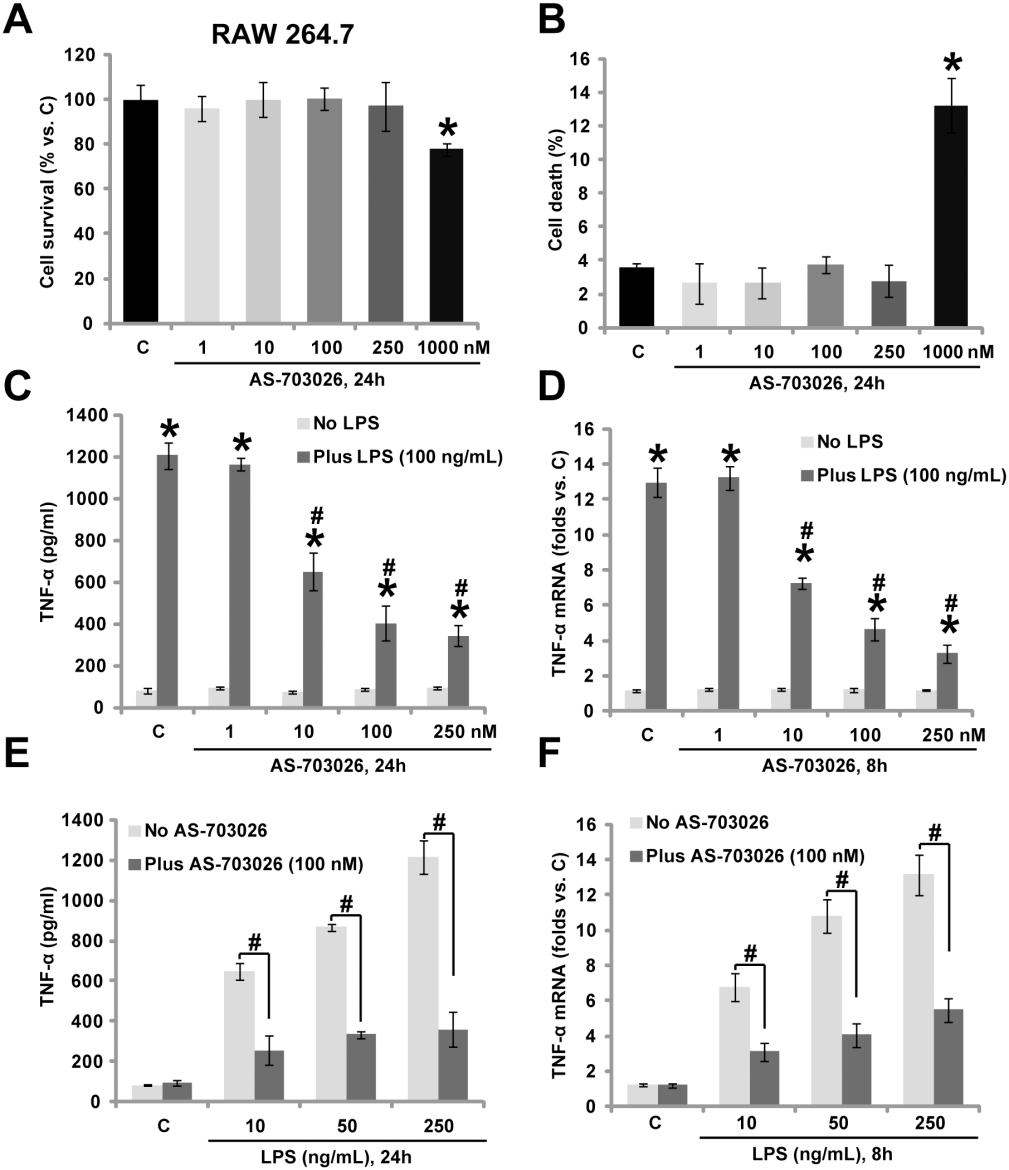
AS-703026 inhibits LPS-mediated TNFα production in RAW 264.7 murine macrophages. RAW 264.7 cells were treated with applied concentrations of AS-703026, and cultured for additional 24 h, cell survival was tested by MTT assay (A), while cell death was examined by trypan blue dye assay (B). RAW 264.7 cells were treated with LPS (10–250 ng/mL), or plus indicated AS-703026 co-treatment, TNFα content in conditional medium was tested by ELISA assay after 24 h (C and E); Relative TNFα mRNA expression was examined after 8 h (D and F). “C” stands for medium control group (Same for all figures). The results presented were representative of three independent experiments, for each assay, n = 5 (Same for all figures). Bars stand for means ± SD (Same for all figures).* *p* < 0.05 compared with “C” group (A-D). ^#^ *p* < 0.05 vs. LPS only group (C-F).

In the current study, we aim to understand the potential effect of AS-703026, a novel MEK/ERK inhibitor [16,17], on LPS-induced TNFα production in macrophages. Since AS-703026 displayed cytotoxic effect against multiple established human cancer cells [16,17], we first examined its activity on the survival of cultured macrophages. The murine macrophage RAW 264.7 cells were treated with indicated concentrations of AS-703026 for 24 h. MTT cell survival (Fig 1A) assay and trypan blue cell death assay (Fig 1B) results demonstrated that AS-703026 was safe to RAW 264.7 cells. Significantly, as demonstrated in Fig 1C, AS-703026 at 10–250 nM dose-dependently inhibited LPS (100 ng/mL)-induced TNFα production in RAW 264.7 cells. Further, TNFα mRNA expression by the LPS treatment was also inhibited (Fig 1D). AS-703026-mediated inhibition on TNFα expression/production was also seen in response to other LPS concentrations in RAW 264.7 cells (Fig 1E and 1F). Collectively, these results demonstrate that AS-703026 inhibits LPS-induced TNFα production in RAW 264.7 macrophages.

### 3.2. AS-703026 inhibits LPS-induced TNFα production in murine bone marrow-derived macrophages (BMDMs)

**Fig 2:**
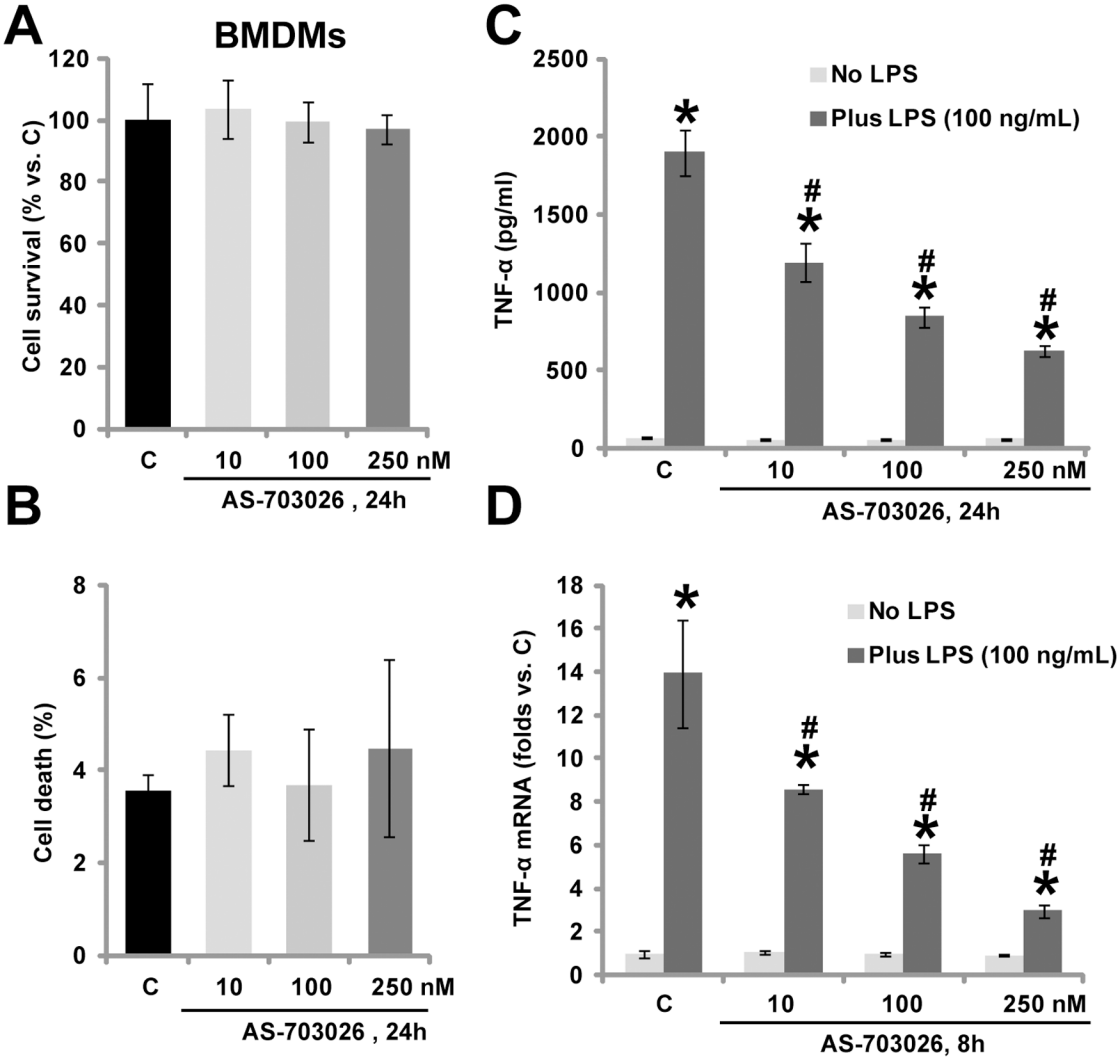
AS-703026 inhibits LPS-induced TNFα production in murine BMDMs. Primary cultured murine BMDMs were treated with applied concentration of AS-703026, and were cultured for additional 24 h, cell survival and cell death were tested by MTT assay (A) and trypan blue dye assay (B), respectively. Murine BMDMs were treated with LPS (100 ng /mL), or plus indicated concentrations of AS-703026, TNFα content in conditional medium (C, after 24 h) and relative TNFα mRNA expression in the BMDM cells (D, after 8 h) were tested. * *p* < 0.05 compared with “C” group (C-D). ^#^ *p* < 0.05 vs. LPS only group (C-D).

The potential effect of AS-703026 on murine BMDMs was also tested. AS-703026 at tested concentrations (10–250 nM) was again safe to primary murine BMDMs (Fig 2A and 2B). Cell viability (Fig 2A) and trypan blue staining (Fig 2B) was almost unchanged after applied AS-703026 treatment. LPS treatment (100 ng/mL) in BMDMs induced significant TNFα production, which was remarkably inhibited by AS-703026 (10–250 nM) co-treatment (Fig 2C). The activity of AS-703026 was dose-dependent, and was most significant at 250 nM (Fig 2C). Real-time PCR results demonstrated that LPS-induced TNFα mRNA expression was also inhibited by AS-703026 in BMDMs (Fig 2D). Together, these results demonstrate that AS-703026 inhibits LPS-induced TNFα production in primary murine BMDMs.

### 3.3. AS-703026 inhibits LPS-mediated TNFα production in *ex-vivo* cultured PBMCs of COPD patients

**Fig 3:**
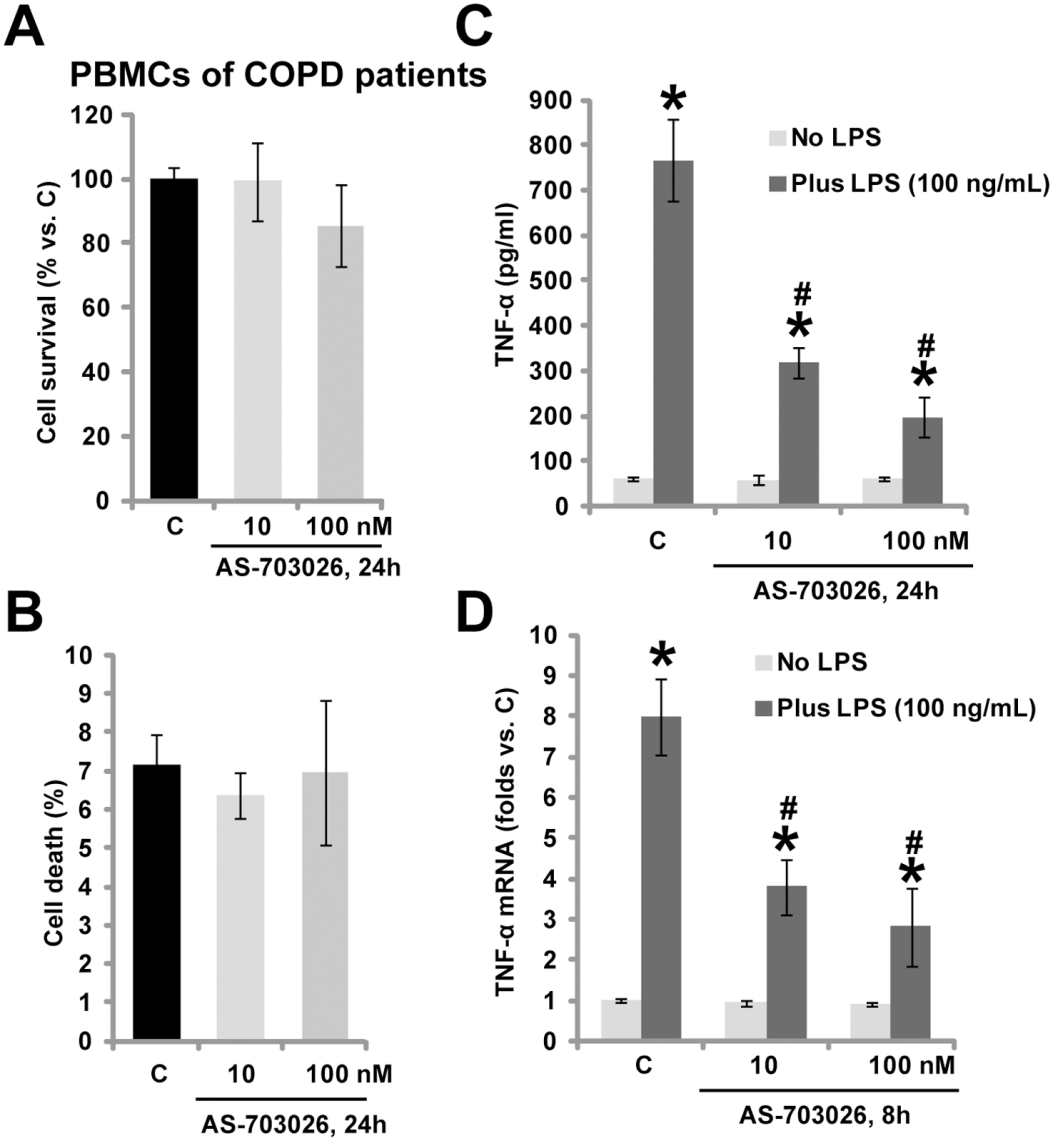
AS-703026 inhibits LPS-mediated TNFα production in *ex-vivo* cultured PBMCs of COPD patients. *Ex-vivo* cultured PBMCs form COPD patients were treated with applied concentration of AS-703026 (10/100 nM), cells were cultured for additional 24 h, cell survival and cell death were tested by MTT assay (A) and trypan blue dye assay (B), respectively. The PBMCs were treated with LPS (100 ng/mL), or plus AS-703026 (10/100 nM), TNFα production (C) and mRNA expression (D) were tested similarly. * *p* < 0.05 compared with “C” group (C-D). ^#^ *p* < 0.05 vs. LPS only group (C-D).

The potential role of AS-703026 in primary human monocytes was also analyzed. We *ex-vivo* cultured primary PBMCs from COPD patients (See methods). Again, we failed to detect any cytotoxic effects after applied AS-703026 treatment in above patients’ PBMCs (Fig 3A and 3B). Importantly, LPS-mediated TNFα production in the PBMCs was dramatically inhibited by AS-703026 co-treatment (Fig 3C). Further, TNFα mRNA expression in response to LPS was also inhibited (Fig 3D). Thus, in line with the macrophage data, in COPD patients’ monocytes, LPS-mediated TNFα production was again inhibited by AS-703026. We repeated those experiments in PBMCs of four other COPD patients, and similar results were obtained.

### 3.4. AS-703026 blocks LPS-induced MEK/ERK activation in murine macrophages and human monocytes

**Fig 4:**
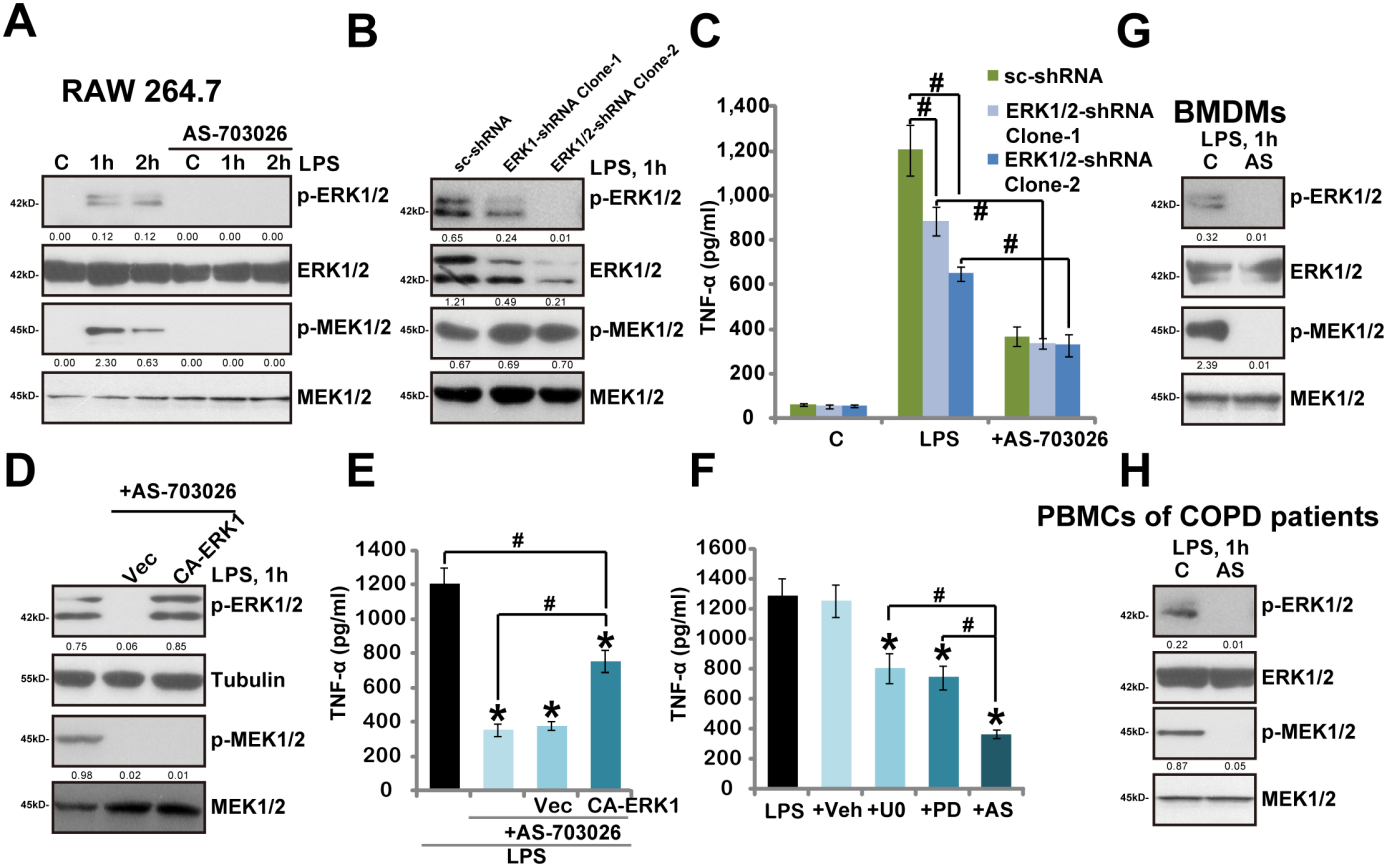
AS-703026 blocks LPS-induced MEK/ERK activation in murine macrophages and human monocytes. RAW 264.7 cells, BMDMs and PBMCs of COPD patients were treated with LPS (100 ng/mL), or plus AS-703026 (AS, 100 nM), after applied time, phosphorylated (p-) and regular MEK1/2 and ERK1/2 were tested by Western blots (A, G and H). Scramble shRNA (“sc-shRNA”)- or ERK1/2 shRNA (-1/-2)-expressing stable RAW 264.7 cells, pre-treated with AS-703026 (100 nM, 1 h), were stimulated with LPS (100 ng/mL), expressions of MEK1/2-ERK1/2 were tested after 1 h (B), TNFα production was tested by ELISA after 24 h (C). Scramble RAW 264.7 cells expressing empty vector or CA-ERK1 were stimulated with LPS (100 ng/mL), or plus AS-703026 (100 nM), expressions of indicated proteins were tested by Western blots after 1 h (D), TNFα production was tested after 24 h (E). TNFα production in RAW 264.7 cells treated with LPS (100 ng/mL, 24 h) or plus MEK/ERK inhibitors U0126 (U0, 5 μM), PD98059 (PD, 5 μM) or AS-703026 (AS, 100 nM) was tested (F). Kinase phosphorylation (vs. regular kinase) was quantified (A, B, D, G and H). ERK1/2 expression (vs. regular MEK1/2) was also quantified (B). * *p* < 0.05 compared with LPS only group (E and F). ^#^ *p* < 0.05 (C, E and F).

Above results showed that AS-703026 inhibited LPS-induced TNFα production in mouse macrophages and human monocytes. The underlying signaling mechanisms were also tested. As expected, LPS treatment in RAW 264.7 cells induced significant MEK/ERK activation/phosphorylation, which was almost completely blocked by AS-703026 co-treatment (Fig 4A). Interestingly, our results implied that AS-703026-mediteaed inhibition on TNFα was not solely dependent on MEK/ERK blockage. First, we showed that AS-703026 was still functional (anti-TNFα) in ERK1/2 shRNA-silenced RAW264.7 cells, where LPS was less effective (Fig 4B and 4C). As expected, MEK phosphorylation was not affected by ERK1/2 shRNA (Fig 4B). Further, exogenous expression of constitutively-active (CA) ERK1, which restored ERK (but not MEK) activation in AS-703026-treatd RAW264.7 cells (Fig 4D), only partially reinstated TNFα production by LPS (Fig 4E). Third, AS-70302 was more potent than traditional MEK/ERK inhibitors, including PD98059 and U0126, in inhibiting LPS-mediated TNFα production (Fig 4F). Note that LPS-induced MEK/ERK activation was completely blocked the two traditional MEK/ERK inhibitors (Data not shown). In primary mouse BMDMs and human PMBCs, LPS-induced MEK/ERK activation was again completely blocked by AS-703026 (Fig 4G and 4H). All these data indicate that AS-703026-induced activity against TNFα production appears not solely dependent on MEK/ERK blockage.

### 3.5. AS-703026 inhibits LPS-induced NFκB activation

**Fig 5:**
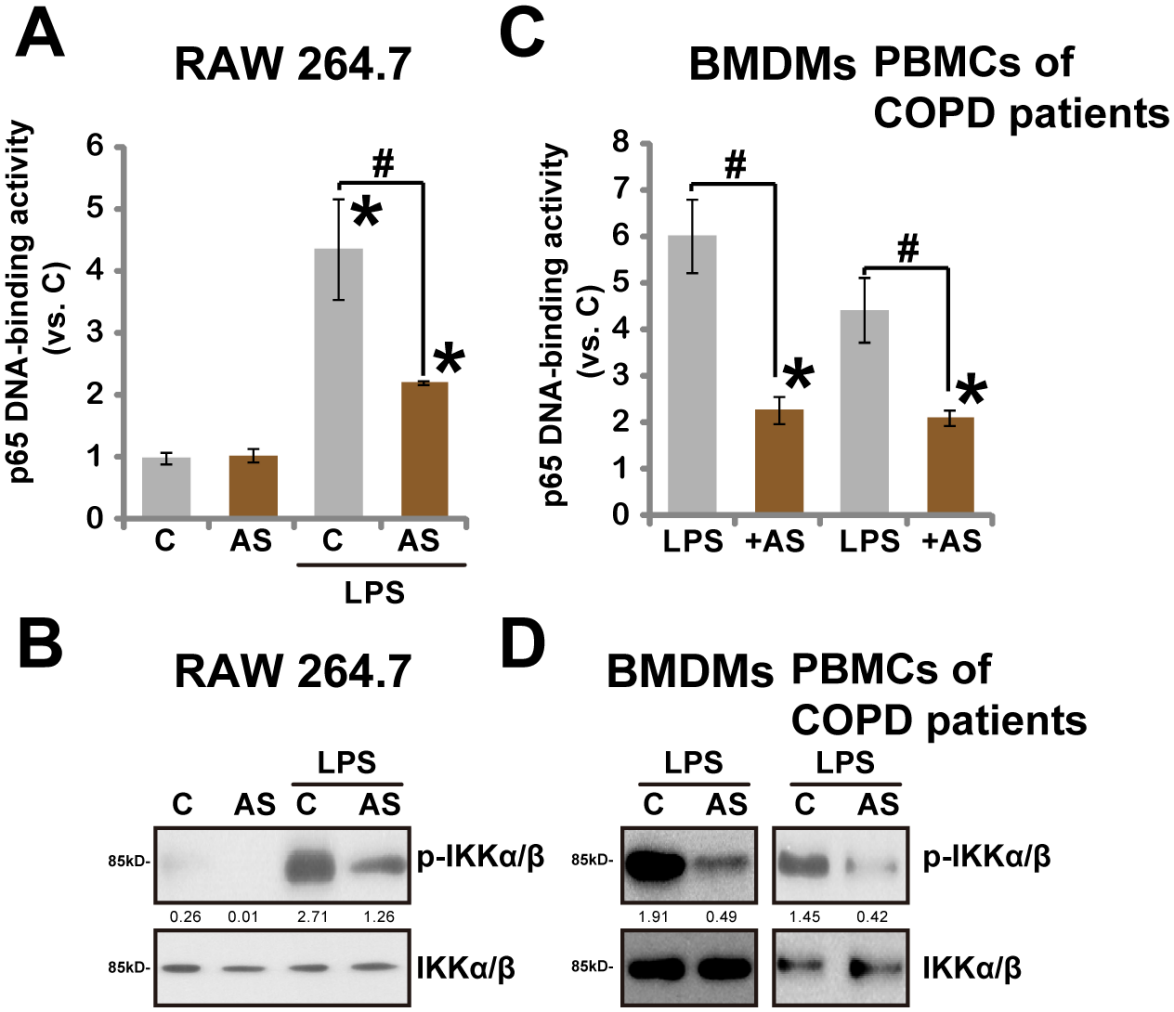
AS-703026 inhibits LPS-induced NFκB activation. RAW 264.7 cells, BMDMs and COPD patients’ PBMCs were treated with LPS (100 ng/mL, 2 h), or plus AS-703026 (AS, 100 nM), NFκB (p65) DNA-binding activity was analyzed, and the values were normalized to “C” group (A and C), p- and regular IKKα/β were tested by Western blots (B and D). IKKα/β phosphorylation (vs. regular IKKα/β) was quantified (C and D). * *p* < 0.05 compared with “C” group (A and C). ^#^ *p* < 0.05 vs. LPS only group (A and C).

Above results indicate that AS-703026-exerted inhibition on TNFα production is not solely dependent on MEK/ERK blockage. NFκB signaling is another important mediator of TNFα production by LPS [6]. Thus, we studied the effect of AS-703026 on LPS-induced NFκB activation in macrophages/monocytes. To our surprise, we found that LPS-induced NFκB activation, tested by p65 DNA-binding assay and Western blot assay of p-IKKα/β (Ser176/180), was inhibited by AS-703026 in RAW264.7 cells (Fig 5A and 5B). Similar NFκB inhibition by AS-703026 was also seen in BMDMs and COPD patients’ monocytes (Fig 5C and 5D). Note that LPS-induced NFκB activation was not affected by two traditional MEK/ERK inhibitors PD98059 and U0126 (Data not shown). Meanwhile, CA-ERK1 failed to restore NFκB activation after AS-703026 treatment (Data not shown). Thus, AS-703026-mediated NFκB inhibition is unlikely associated with MEK/ERK blockage. Together, we demonstrate that AS-703026 inhibits LPS-induced NFκB activation, which could also be responsible, at least in part, for its effect against LPS-induced TNFα production in macrophages/monocytes.

### 3.6. AS-703026 inhibits LPS-induced endotoxin shock and TNFα production in BALB/c mice

**Fig 6:**
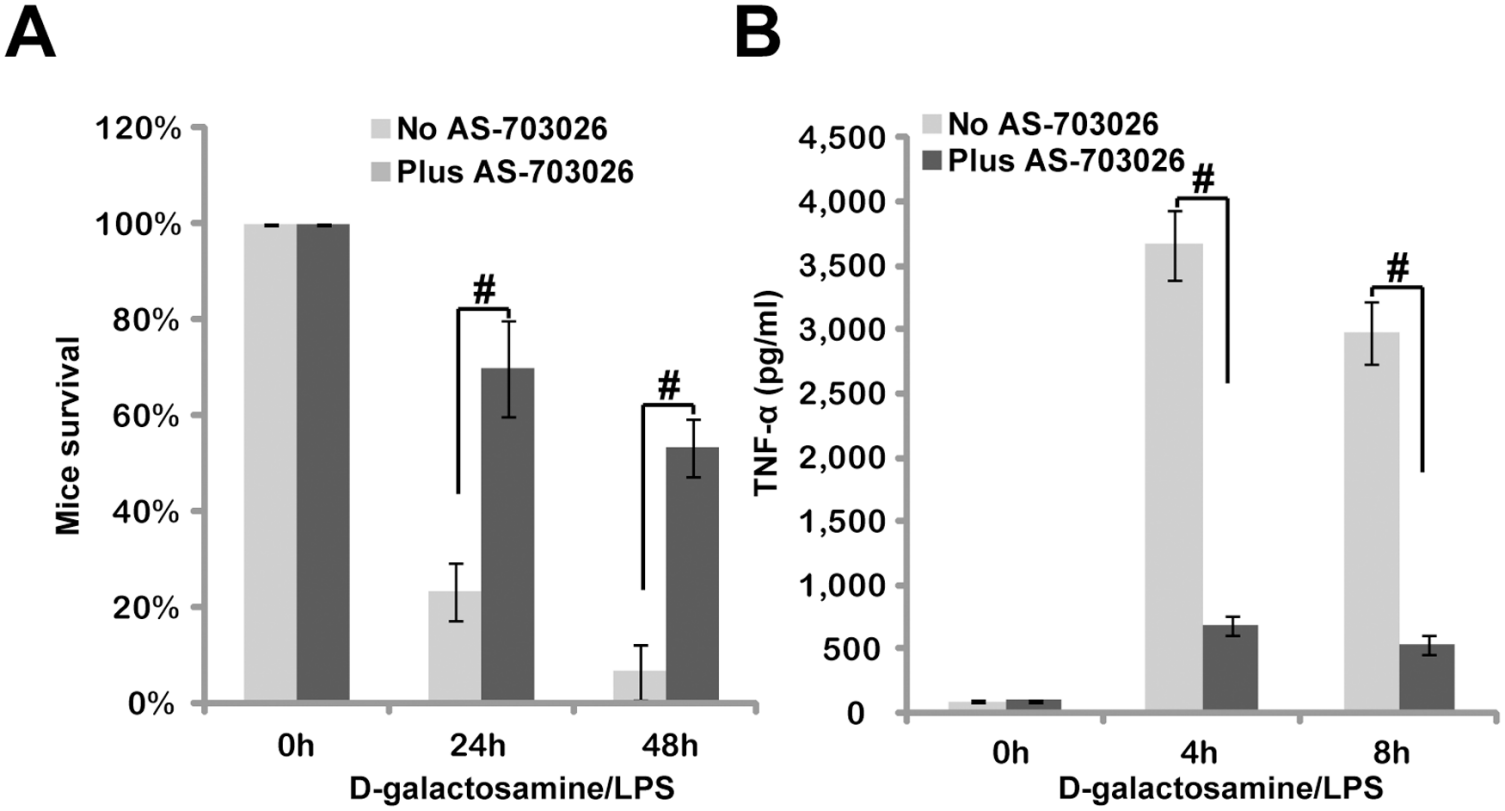
AS-703026 inhibits LPS-induced TNFα production and endotoxin shock in BALB/c mice. BALB/c mice (4–6 weeks old, 6 mice per group) were injected *i*.*p*. with LPS (30 mg/kg body weight) and D-galactosamine (300 mg/kg body weight), or plus oral gavage of AS-703026 (30 mg/kg mice body weight), mice death was recorded 24 h and 48 h after LPS administration (A), tail vein serum samples were collected 4 h and 8 h after LPS stimulation, TNFα content was determined by ELISA (B). *In vivo* experiments were repeated three times. ^#^ *p* < 0.05.

Finally, the potential role of AS-703026 on LPS-induced inflammatory response was tested *in vivo*. BALB/c mice were inoculated *i*.*p*. with LPS (30 mg/kg body weight) plus D-galactosamine (300 mg/kg), the latter is a hepatotoxic transcriptional inhibitor which sensitizes the cytotoxic effects of TNFα [22]. We demonstrated that LPS administration induced septic shock within 24 h to 48 h, causing dramatic mortality in BALB/c mice (Fig 6A). Importantly, co-administration of AS-703026 (30 mg/kg) remarkably protected mice from LPS/D-galactosamine, and endotoxin shock was largely inhibited (Fig 6A). The concentration of AS-703026 *in vivo* was determined based on previous studies [16,17], where no significant toxicities were observed to tested animals. Over 50% of AS-703026-co-administrated mice were still alive 48 h after LPS/D-galactosamine challenge (Fig 6A). These surviving mice were observed for additional 5–6 days, and no late occurring toxic effects or mortality were observed (Data not shown). The effect of AS-703026 on TNFα production was also analyzed *in vivo*. ELISA results analyzing mice tail vein serum demonstrated that AS-703026 significantly inhibited LPS/D-galactosamine-induced TNFα production in BALB/c mice (Fig 6B). Together, we showed that AS-703026 oral administration significantly inhibited LPS-induced TNFα production *in vivo*, and protected mice from endotoxin shock.

## Discussions

In the current study, we showed that AS-703026, a novel MEK/ERK inhibitor, dramatically inhibited LPS-induced TNFα production in macrophages (primary cells and RAW 264.7 cells). Meanwhile, TNFα production in LPS-stimulated PBMCs of COPD patents was also inhibited by AS-703026. At the molecular level, we showed that AS-703026-exerted anti-TNFα activity was likely mediated through MEK/ERK-dependent and -independent mechanisms.

AS-703026 is a potent MEK/ERK inhibitor [16,17]. Here we demonstrated that AS-703026 blocked MEK/ERK activation by LPS in murine macrophages and monocytes of COPD patients. However, our results indicated that AS-703026-medaited anti-TNFα activity appeared not solely dependent on MEK/ERK blockage. First, we found that AS-703026 was more potent than traditional MEK/ERK inhibitors (PD98059 and U0126) in repressing LPS-mediated TNFα production. Further, although exogenous CA-ERK1 restored ERK activation in AS-703026-treated RAW 264.7 cells, it only partially reinstated TNFα production by LPS. Third, AS-703026 could still inhibit LPS-induced TNFα production in ERK1/2-depleted RAW 264.7 cells. Thus, other mechanisms besides MEK/ERK inhibition could also be responsible for its activity in monocytes/macrophages.

As a matter of fact, one important finding of this study is that LPS-induced NFκB activation was also inhibited by AS-703026 in macrophages and COPD patients’ PMBCs. Thus, AS-703026-mediated anti-TNFα activity could also be due to its effect on NFκB signaling. At the current stage, the detailed mechanisms of NFκB inhibition by AS-703026 were still under investigation. However, it is unlikely that NFκB inhibition by AS-703026 is the consequence of MEK/ERK blockage. Since restoring ERK activation by exogenously introducing CA-ERK1 had no effect on NFκB activation in RAW264.7 cells (Data not shown). Also, traditional MEK/ERK inhibitors (PD98059 and U0126), or shRNA-mediated silencing of ERK1/2, were ineffective on NFκB activation in LPS-treated macrophages/monocytes (Data not shown). Thus, NFκB inhibition is possibly an unique effect by this novel MEK/ERK inhibitor, and the detailed signaling mechanisms warrant further investigations.

There are several advantages using this novel MEK/ERK inhibitor. AS-703026 displayed superior efficiency in inhibiting LPS-induced TNFα production, more potently than traditional MEK/ERK inhibitors (PD9809 and U0126). *In vitro*, AS-703026 at nM concentrations could significantly inhibit TNFα production in both primary and established macrophages/monocytes. Further, besides exerting MEK/ERK blockage activity, AS-703026 could also inhibit LPS-induced NFκB activation, which at least in part explains its superior activity. The another advantage of using this novel MEK/ERK inhibitor is its oral availability. As a matter of fact, here we demonstrated that AS-703026 oral administration significantly inhibited LPS-induced endotoxin shock; Mice with AS-703026 administration were protected from LPS challenge.

## Conclusions

Together, these results demonstrate that AS-703026 *in vitro* inhibits LPS-induced TNFα production in macrophages and COPD patients’ monocytes, and *in vivo* protects mice from LPS-induced endotoxin shock. These results suggest that AS-703026 could be further studied as a useful anti-inflammatory therapy for COPD patients.
